# Testing Phylogeographic Hypotheses in *Mepraia* (Hemiptera: Reduviidae) Suggests a Complex Spatio-Temporal Colonization in the Coastal Atacama Desert

**DOI:** 10.3390/insects13050419

**Published:** 2022-04-29

**Authors:** Ricardo Campos-Soto, Evelyn Rodríguez-Valenzuela, Gabriel Díaz-Campusano, Dusan Boric-Bargetto, Álvaro Zúñiga-Reinoso, Franco Cianferoni, Fernando Torres-Pérez

**Affiliations:** 1 Escuela de Ciencias Agrícolas y Veterinarias, Universidad Viña del Mar, Viña del Mar 2572007, Chile; 2 Instituto de Biología, Facultad de Ciencias, Pontificia Universidad Católica de Valparaíso, Valparaíso 2373223, Chile; evelyn.rodriguez.v@pucv.cl (E.R.-V.); gabrieldiazcampusano@gmail.com (G.D.-C.); dusan.boric@pucv.cl (D.B.-B.); franco.cianferoni.a@gmail.com (F.C.); fernando.torres@pucv.cl (F.T.-P.); 3 Institute for Zoology, University of Cologne, 50674 Cologne, Germany; azunigar@uni-koeln.de

**Keywords:** *Mepraia* species, Biogeography of *Mepraia*, Plio-Pleistocene climate change, Andes uplift, phylogeographic hypotheses, diversification, colonization, phylogeny, blood-sucking bug, vector-borne disease

## Abstract

**Simple Summary:**

*Mepraia* is a blood-sucking bug endemic to Chile and a vector of the parasite that causes Chagas disease. Different colonization routes have been suggested for this bug; therefore, we tested different colonization routes using DNA sequences and bioinformatics approaches to select the most probable route. Our results suggest that, after the split of *Triatoma*, *Mepraia* divided into two main groups ~2.1 Mya. The northern group would have speciated between 1.7–1.4 Mya, giving rise to *M. parapatrica, M. gajardoi* and to a new, still undescribed lineage (*Mepraia* sp.). The southern group formed *M. spinolai* ~1.68 Mya. We suggest that *Mepraia* originated from the north-central Andes due to the last Andes uplift and hyperaridity. The hyperarid cycle would have separated the southern and northern groups. Then, within the northern group, colonization would have occurred from the centre to the north and south through corridors influenced by Pleistocene climatic changes. The habitat colonized by the southern clade led to only one species (*M. spinolai*). Fluctuations in climatic changes probably influenced speciation strongly in this kissing bug in the Atacama Desert.

**Abstract:**

*Mepraia* is a genus (Triatominae) endemic to Chile and a vector of *Trypanosoma cruzi*. Alternative phylogeographic hypotheses have been suggested for *Mepraia*. We tested different colonization routes hypothesized using mitochondrial sequences and phylogeographic approaches to select the best-supported hypothesis. Our results suggest that, after the split from the sister genus *Triatoma* at ~4.3 Mya, *Mepraia* formed two main clades at ~2.1 Mya. The northern clade diverged from *Mepraia* sp. ~1.7 Mya, giving rise to *M. parapatrica* and *M. gajardoi* about ~1.4 Mya. The southern clade originated *M. spinolai* ~1.68 Mya. We suggest that *Mepraia* had an origin in the north-central Andes along with orogenic processes, reinforced by hyperaridity during the Pliocene. The hyperarid cycle would have separated the southern and northern clades. Then, in the northern clade, dispersal occurred north and south from the centre through corridors during the Pleistocene Climatic Oscillations. Climate changes may have induced a major speciation process in the Atacama Desert, while the more homogeneous habitat colonized by the southern clade led to only one, but structured, species.

## 1. Introduction

**Figure 1:**
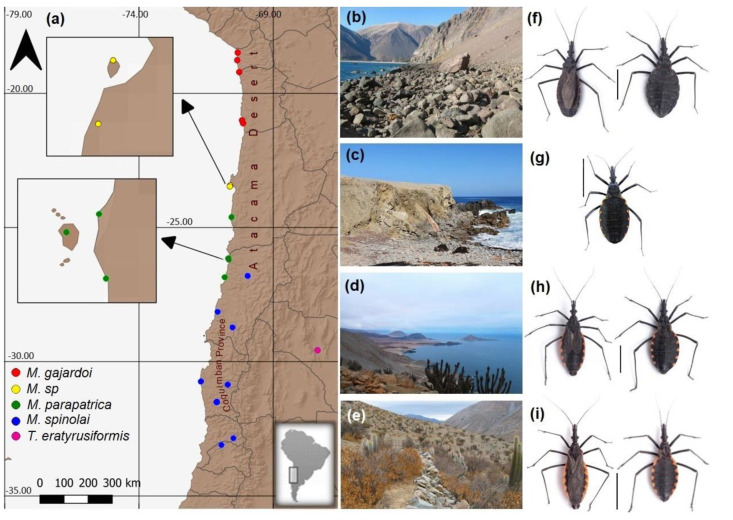
(**a**) Collection sites of *Mepraia* species with the biogeographic characteristics of the current habitats. (**b**) Sampling site of *M. gajardoi* in the extreme north of the coastal Atacama Desert (Caleta Vitor), photograph by Ricardo Campos-Soto. (**c**) Sampling site of *M.* sp. on the coast of the Mejillones Peninsula (Santa María Island) in the Atacama Desert, photograph by Ricardo Campos-Soto. (**d**) Habitat of *M. parapatrica* in the Pan de Azúcar National Park in the Atacama Desert, photograph by Ricardo Campos-Soto. (**e**) Sampling site of *M. spinolai* in the interior valley of central Chile in the Coquimban Biogeographical Province [17], photograph by Ricardo Campos-Soto. (**f**) Adult *M. gajardoi*, male on the left and female on the right, photographs by Vicente Valdés. (**g**) Female adult *M.* sp., photograph by Ricardo Campos-Soto (**h**) Adults of *M. parapatrica*, male on the left and female on the right, photographs by Vicente Valdés. (**i**) Adult *M. spinolai*, male on the left and female on the right, photographs by Vicente Valdés. The vertical black bar represents one centimetre.

One of the most important historical events that shaped the current biodiversity in South America is, without a doubt, the Andes uplift, because this huge mountain range is a clear east–west barrier and because both climate and landscape changed as a consequence of the uplift, producing high diversification in this continent [1]. These landscape changes allowed some taxa to disperse to other places because new niches were available [2], whereas other taxa were enclosed in small, suitable patches [3], possibly triggering species pump events. The origin and diversification of the tribe Triatomini seem to be linked with the events explained above, which allowed the colonization of southern South America from a Mesoamerican origin [4]. The genus *Mepraia* is a Triatomini with a sub-Andean distribution and could be one of the latest groups to diversify. This taxon is endemic to the arid and semiarid regions of Chile; it is known widely because *Mepraia* species are blood-sucking bugs responsible for the transmission of *Trypanosoma cruzi* in the wild cycle, causing Chagas disease [5,6]. *Mepraia* is composed of three species: *M. gajardoi*, *M. parapatrica* and *M. spinolai. M. gajardoi* and *M. parapatrica* have been found exclusively in the coastal Atacama Desert between 18°30′ S and 26°51′ S [7,8], whereas *M. spinolai* inhabits from 26° to 34° S, mainly in the interior of valleys and, to a lesser extent, in coastal areas [7,8] (Figure 1). In addition to these species, a new coastal lineage has been reported in Chile (*M*. sp. from now on), which inhabits between the distributions of *M. parapatrica* and *M. gajardoi* (23°25′ to 23°28′ S) [9]. The coastal species inhabit landscapes with low vegetation under stones and in rock crevices, associated with marine bird nests, rodent burrows and lizards [9,10]. Although each of the *Mepraia* species forms well-supported clades, the phylogenetic relationships between these lineages are still unresolved, leading to controversy regarding the colonization routes of *Mepraia* species in Chile [7,9]. One of the colonization hypotheses of *Mepraia* states that, due to the divergence of an ancestral population by the uplift of the Andes Range, *Mepraia* would have separated from the eastern slope species *Triatoma eratyrusiformis* and *T. breyeri*. Later, the process of speciation in *Mepraia* would have followed latitudinal colonization from north to south [8]. According to this hypothesis, the most northerly species *M. gajardoi* would be sister to the remaining species (*M. parapatrica* and *M. spinolai*) and descended from the most ancient ancestor. However, this hypothesis is not consistent with the time-calibrated phylogeny, which suggests that the most southerly species *M. spinolai* is sister to the remaining species (*M. gajardoi*, *M. parapatrica* and *M.* sp.) [7,9]. Therefore, new alternative colonization scenarios need to be evaluated. In this study, we used sequences of three mitochondrial genes to assess spatio-temporal scenarios that could explain the historical colonization routes of *Mepraia*.

## 2. Materials and Methods

### 2.1. Samples, Localities and Obtaining Mitochondrial Gene Sequences

We used a set of DNA samples extracted from previous studies [7,9], which were stored at −80 °C. These samples were from localities that covered the distribution ranges of all *Mepraia* species, and a *T. eratyrusiformis* sample used as outgroup (Figure 1, Table S1). Segments of 631 bp for cytochrome oxidase subunit I (COI, called COIA) and 563 bp for NADH dehydrogenase subunit 4 (ND4) of each selected sample were amplified by PCR using the polymerase SapphireAmp^®^ fast PCR Master Mix (Takara, Kusatsu shi, Japan). We used the primers COIF (5′-CCTGCAGGAGGAGGAGAYCC) and CO10R (5′-TAAGCGTCTGGGTAGTCTGARTAKCG) used in [11] for the COIA segment, and the primers ND4-F (5′-TCAACATGAGCCCTTGGAAG) and ND4-R (5′-TAATTCGTTGTCATGGTAATG) described in [12] for the ND4 segment. The same cycling conditions were used to amplify the COIA and ND4 segments using a Bioer model TC-96/G/H(b)C LifeEco^®^ thermocycler (Hangzhou, China). The cycling conditions were 30 s at 94 °C, followed by 40 cycles of 94 °C for 30 s, 55 °C for 30 s and 72 °C for 1 min, according to the manufacturer’s instructions. Amplification was confirmed by electrophoresis in 1% agarose gels. The sequencing of both strands was performed by Macrogen, Inc. (Seoul, Korea) using the same PCR primers. Sequences were edited to obtain consensus sequences using BioEdit 7.0.4.1 [13] and aligned using Clustal W [14] incorporated in BioEdit. After alignment, sites that showed nucleotide substitutions were re-examined by the visual inspection of each individual’s raw chromatogram. The COI and ND4 gene sequences were deposited in GenBank with the accession numbers MN117859-MN117888. The samples sequenced for each locality and GenBank accession numbers are described in Table S1. The COIA and ND4 sequences of each sample were manually concatenated with a previous matrix [9] formed by another COI segment designated as COIB and a cyt b segment, obtaining a final matrix of 2237 pb (Table S1) composed of four segments from three genes. New COIA and ND4 and previous sequences [7,9] were obtained from the same kissing bugs for each locality. The nucleotide substitution model was selected with the Bayesian Information Criterion (BIC) using Smart Model Selection [15] in the ATGC bioinformatics platform [16]. The best model was HKY + G for both COI segments and TN93 + G for the cyt b and ND4 segments.

### 2.2. Phylogenetic Analyses and Estimates of Time of the Most Recent Common Ancestor (TMRCA)

Phylogenetic reconstruction was inferred with the final concatenated matrix (2237 bp), which was partitioned by gene and codon position; trees were linked using BEAUti, implemented in BEAST v.2.6.4 [18]. Phylogenetic relationships and TMRCA were estimated with the Bayesian Markov Chain Monte Carlo (BMCMC) method available in BEAST v.2.6.4 [18]. A Yule model was used as prior, run first using the uncorrelated lognormal relaxed molecular clock, which was calibrated using the mutation rates previously estimated for each gene. A mutation rate of 0.016 substitutions/site/million years (Myr) was used for the COI gene segment (COIA + COIB) [19], 0.023 for cyt b [20] and 0.11 for ND4 [21]. These mutation rates were used as means with a normal distribution in the priors for the molecular clock to estimate the best mutation rates for each segment. The estimated mutation rates (substitutions/site/Myr) were 0.016 for COI, 0.023 for cyt b and 0.047 for ND4. Using these rates and the Yule model as prior, uncorrelated lognormal relaxed and strict molecular clocks were run. The best molecular clock was selected using the Nested Sampling package [22] available in BEAST v.2.6.4. using 100 particles in each model run. This analysis estimated the log Marginal Likelihood (log(ML)) for each model. The log Bayes Factors (log (BF)) were calculated using the log(ML) of each model in the equation: log(BF) = log(ML1) − log(ML2). If log(BF) is greater than 1, the first model is favoured [23]. The best resulting model estimated was the strict molecular clock. With the Yule model and selected molecular clock, two separate MCMC runs of 5 × 10^7^ generations were run using the BEAST program, sampling every 5000 generations. Mixing and convergence were verified based on the effective sample size (ESS) values of each run in Tracer v.1.7 [24]; only ESSs > 500 were accepted in each run. In both runs, the initial 10% of trees were discarded and the rest were combined using LogCombiner. The statistical uncertainty was depicted in values of the 95% Highest Probability Density (HPD) and node support was evaluated with posterior probabilities (pp); only pp > 0.95 were accepted as supported. The consensus tree was calculated with the maximum clade credibility and mean node heights option using TreeAnnotator. The consensus tree was visualized and edited in FigTree v1.4.4. [25].

### 2.3. Test for Phylogeographic Scenarios Using Coalescent Simulations

**Figure 2:**
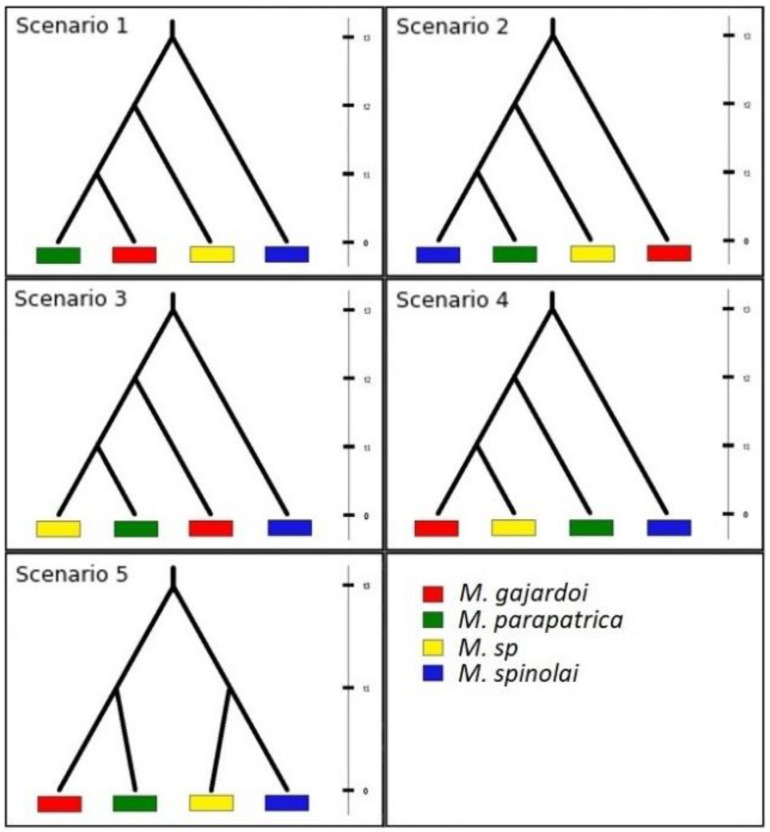
Phylogeographic hypotheses tested in this study. Scenario 1: hypothesis generated by the BMCMC proposed colonization from the Coquimban Biogeographical Province (Figure 1a) to the north, where the most southerly species *M. spinolai* is sister to the northern clade formed by *M. gajardoi*, *M. parapatrica* and *M.* sp, and, within this clade, *M.* sp. is sister to the remaining species *M. gajardoi* and *M. parapatrica* [9]. Scenario 2 represents the colonization hypothesis from north to south proposed by Frias-Lasserre (2010) [8]. Scenario 3: *M. spinolai* is sister to a northern clade that includes *M. gajardoi* as sister to the remaining species *M.* sp. and *M. parapatrica*. Scenario 4 suggests colonization from south to north following a parapatric speciation model, where *M. spinolai* is sister to a clade that includes *M. parapatrica* as sister to the remaining species *M. gajardoi* and *M.* sp. Scenario 5 shows a southern clade formed by *M. spinolai* as sister to *M.* sp. and a northern clade formed by *M. gajardoi* and *M. parapatrica*.

In order to better understand the diversification process of *Mepraia* in Chile, we used the coalescent-based Approximate Bayesian Computation (ABC) method implemented in DIYABC v2.2 [26]. In this approach, different models (scenarios) are compared to judge how well each fits the observed data by generating simulated data using historical and mutational parameters. ABC analyses were conducted on the sequence data of the COIB and cyt b segments of all individuals (Table S1). Based on the phylogenetic results, we considered four groups (*M. gajardoi*, *M*. sp., *M. parapatrica* and *M. spinolai*) and constructed five phylogeographic scenarios. Scenario 1 represents the hypothesis generated by the BMCMC method [9] that suggests colonization from the Coquimban Biogeographical Province (centre-south) [17] to the north (Figure 1a). In this scenario, the most southerly species *M. spinolai* is sister to the northern clade formed by *M. gajardoi*, *M. parapatrica* and *M.* sp., and, within this northern clade, *M*. sp. is sister to the remaining species *M. gajardoi* and *M. parapatrica* (Figure 2). Scenario 2 represents the hypothesis of colonization from north to south proposed by Frías-Lasserre (2010) [8] (Figure 2). In scenario 3 *M. spinolai* is sister to the northern clade; however, within this northern clade, *M. gajardoi* is sister to the remaining species *M.* sp. and *M. parapatrica* (Figure 2). Scenario 4 suggests colonization from south to north following a parapatric speciation model, where *M. spinolai* is sister to the northern clade, which includes *M. parapatrica* as sister to the remaining species *M. gajardoi* and *M*. sp. (Figure 2). Scenario 5 proposes a southern clade formed by *M. spinolai* and *M*. sp. and a northern clade formed by *M. gajardoi* and *M. parapatrica* (Figure 2). We used the estimations calculated in this study by the BMCMC method to set the divergence times for the scenarios (Table S2), and the population sizes were set as constant, as suggested by [7]. We used HKY as the mutational model and a mutation rate of 1.00 × 10^−8^–5.00 × 10^−8^ per site per generation calculated according to the mutation rate of mitochondrial markers (10^−2^ subs/site/my) and a generation time of one year for *Mepraia* species (Table S2). For each scenario, we simulated 1,000,000 datasets and performed pre-evaluations to confirm that our models were suitable for comparison against the observed data. The best scenario was chosen by comparing the posterior probabilities (pp), which were calculated using a logistic regression approach on 1% of the simulated datasets closest to the observed dataset.

### 2.4. Test of Phylogenetic Hypotheses Using Bayes Factor

The phylogeographic scenarios were also compared using BEAST v.2.6.4 to provide more evidence on the diversification process of *Mepraia*. With the final concatenated matrix (COI, cyt b and ND4), five files (xml) were formed with different phylogenetic relationships congruent with scenarios 1 to 5 (Figure 2) using the option “Add Prior” in BEAUti and keeping all other parameters unchanged. Each scenario was run separately with 100 particles and log(ML) was calculated using the Nested Sampling package [22] available in BEAST v.2.6.5. Paired comparisons between scenarios were performed with log(BF), which was calculated with log(ML); if log(BF) is larger than 1, the first scenario is favoured [23]. We assumed the topology generated by the BMCMC method as the first scenario, which was compared with the other scenarios.

## 3. Results

### 3.1. Phylogenetic Relationships and Divergences Time into Mepraia Species

**Figure 3:**
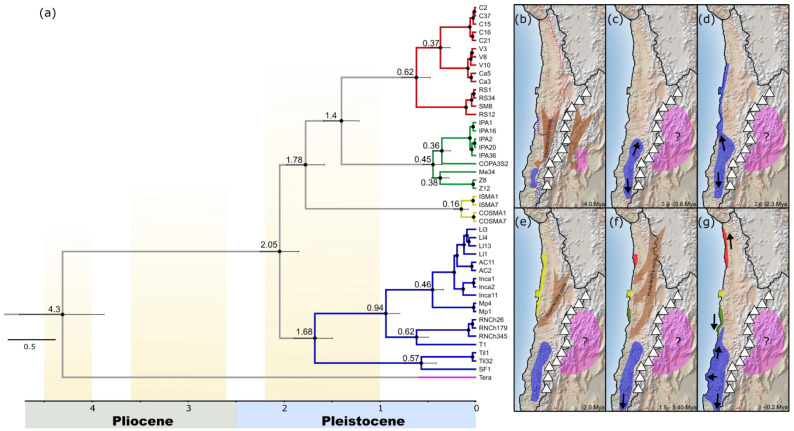
Most probable colonization scenario of *Mepraia* species inferred in BEAST and DIYABC using mitochondrial gene sequences (COI, cyt b and ND4). (**a**) Bayesian consensus tree with divergence times. The numbers at the nodes are the divergence times in millions of years; the black bars at the nodes show the 95% HPD for node age. The black circles at the nodes indicate node support above 0.95 pp. The colour coding of the branches and areas in the map corresponds to the species shown in Figure 1. The timing of the hyperarid cycles is shown in the yellowish shaded area on the tree according to [27] and as brown arrows on the map. (**b**–**g**) illustrate the consecutive biogeographical events. The triangles on the maps illustrate the Andes Range after the last uplift. The red and blue dotted lines on map (**b**) delimit the Atacama and Coquimban Biogeographical Provinces, respectively, according to [17]. The black arrows on the maps indicate the colonization direction.

The phylogenetic BMCMC analyses showed that *Mepraia* split from the Argentinian taxon about 4.3 Mya (HPD: 3.88–4.76). *Mepraia* species were clustered in two supported main clades north and south (Figure 3a). The northern clade was divided into three supported subclades that included *M. gajardoi*, *M. parapatrica* and *M*. sp.; all occurred along the long coastal strip of northern Chile. The southern clade contained only *M. spinolai* (Figure 3a). The estimated TMRCA between the two main clades was about 2 Mya (HPD: 1.85–2.25). Within the northern clade, the estimated TMRCA for *Mepraia* species was 0.62 Mya (HPD: 0.48–0.78) for *M. gajardoi*, 0.45 Mya (HPD: 0.36–0.55) for *M. parapatrica* and 0.16 Mya (HPD: 0.08–0.23) for *M.* sp. *M. gajardoi* was recovered as a sister taxon of *M. parapatrica* and the divergence time of these sister groups was 1.4 Mya (HPD: 1.22–1.59). The TMRCA for the southern *M. spinolai* clade was 1.68 Mya (HPD: 1.5–1.9).

### 3.2. Test of Phylogeographic Hypotheses with Coalescent Simulations

**Figure 4:**
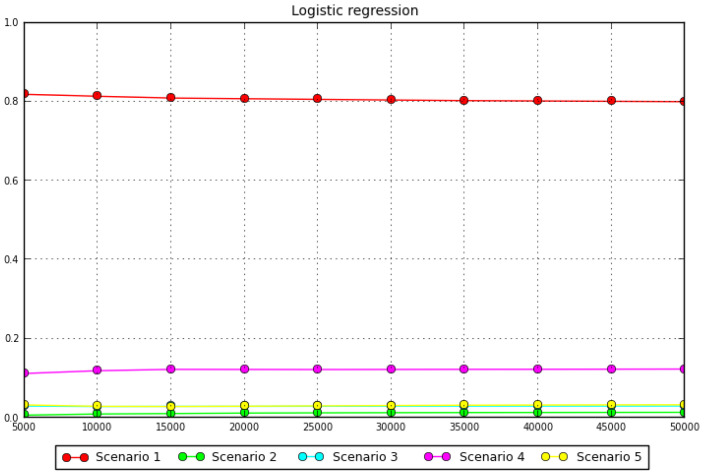
Graph of the posterior probabilities of each scenario estimated using a logistic regression approach.

Approximate Bayesian Computation analyses showed that the scenario with the highest posterior probability was scenario 1, with pp = 0.8 (0.7891–0.8490), followed by scenario 4, pp = 0.112 (0.0868–0.1374), scenario, 5 pp = 0.033 (0.0194–0.0455), scenario 3, pp = 0.0297 (0.0217–0.0378), and finally scenario 2, pp = 0.0066 (0.0017–0.0116) (Figure 4).

### 3.3. Test of Phylogenetic Hypotheses

Paired comparisons between the phylogenetic scenarios showed scenario 1 as the best phylogenetic model. The calculated log(BF) for scenario 1 had positive values above 8.8 in all comparisons, which can be interpreted as overwhelming support in favour of scenario 1. The estimated log(ML) and paired comparisons between scenarios using log(BF) are given in Table 1. The calculated standard deviations were 1.2 for all estimated log(ML).

## 4. Discussion

The effects of the climate changes in South America after the Andes uplift and, in particular, the Plio-Pleistocene climate oscillations (PPCO) were notorious; they caused a strong impact on the landscape and biota in different ecoregions of the continent [28,29]. Although the effects on the biota in the desert areas of Chile have been less studied, the PPCO is considered as one of the drivers of the diversification process, generating high endemism of lineages [3,29,30,31,32]. The hypothesis that was most strongly supported by our results shows that the split between *Mepraia* and Argentinean *Triatoma* species (~4.3 Mya, HPD: 3.88–4.76) likely occurred after the last Andes uplift (Figure 3b), as suggested by Frias-Lasserre (2010) [8]; our results partially support this idea. The last uplift of the Andes was estimated at ~5–3 Mya during the late Pliocene [33]; however, during the Miocene, the Andes already had an altitude similar to today [34]. Therefore, this divergence could have been reinforced by the Pliocene climate changes, which made the weather colder and drier [35], even going through a hyperarid period in the Atacama Desert [27] (Figure 3b). These hyperarid or arid periods were also suggested as the cause of divergence for clades of other insects, reinforcing the role of tectonic and climate changes in the separation of main groups [3,30]. This divergent event would have occurred in the north-central Andes between ~26°–30° S, as in *Psectrascelis* darkling beetles [3] and plant species of the genus *Argylia* [36].

After this basal split, the history of *Mepraia* (~2.1 Mya) began during the Plio-Pleistocene (Figure 3d,e). The climate changes were particularly harsh in the Atacama Desert, switching from arid to hyperarid in the last 25 Mya [27]. Today, the Atacama Desert is hyperarid and most insects avoid the core of the Atacama, occupying other environments, such as the Altiplano [3] and/or, like *Mepraia*, the narrow coastal band. From 28° S, the climate becomes more “friendly”, and roughly from here to the south, *M. spinolai* can occupy inland areas. Today, *M. spinolai* has inhabited the most humid area since one of the most important hyperarid periods at the end of the Pliocene. This could have pushed this species to survive in small suitable habitats, definitely separating *M. spinolai* from the northern clade ancestor. Therefore, our results strongly support the colonization of *Mepraia* from the central highland area to the north (Figure 3d), as was expected from the results of several earlier studies [7,9]. This colonization pathway also was suggested for other insects, where the ancestor could have occupied the Coquimban Biogeographical Province (where *M. spinolai* is distributed) and then colonized the northern Atacama during the Pliocene [3,32]. In fact, the Coquimban Biogeographical Province was demonstrated as the central distribution area for *M. spinolai*, with more diversity than the peripheral populations [37]. The distribution pattern of *Mepraia* today nicely represents the effect of hyperaridity on the distribution of these triatomines; the climate cycling during the Plio-Pleistocene surely affected the diversification of *Mepraia*. However, *Mepraia* are fully dependent on other animals to complete their biological cycle. *Mepraia* have opportunistic feeding behaviour, i.e., they mainly feed on the most abundant or available hosts, including mammals, lizards and birds [38,39]. Therefore, the phylogeographic patterns of *Mepraia* are not only affected by abiotic forces [37], but also depend on the status and distribution of the animal populations on which they feed. In other words, the phylogeography of *Mepraia* is also dependent on the availability and distribution of hosts. High genetic structure is expected under these conditions, and populations of *Mepraia* are highly structured [7]. This pattern is congruent with the other blood-sucking bug (*Triatoma infestans*) [40], which is the only species of *Triatoma* in Chile and is sympatric with *M. spinolai* [41]. The Pleistocene Climatic Oscillation (PCO) generated the extension (in humid periods) and contraction (in dry periods) of semiarid vegetation [42,43]. It is well known that the semiarid vegetation harboured vertebrate fauna during the humid periods of the PCO [44,45] that could be a source of food for triatomines that followed the structuring process. Climatic oscillations in the distribution range of the southern clade had a minor impact, creating a more homogeneous habitat [32] that produced fewer splits or speciation processes. This habitat could have favoured the connection of populations, but, even under those favourable conditions, a deep genetic structure was found within *M. spinolai* [7,37]. 

The PCO would have had a greater impact on the northern clade than on the southern clade. This greater impact was expressed as a major diversification between 1.8 and 1.4 Mya, which drove the origin of three species (Figure 3f), each one also genetically quite structured. All intraspecific structuring processes would have occurred in the last 0.9 Mya, which is congruent with the other colonization process in surrounding areas of the Atacama Desert [3,30]. 

The dry period of climatic oscillations in the area of the northern clade would have caused major population structuring, separating populations by hyperarid barriers, the contraction of semiarid vegetation, and connection by corridors [3,30,31,36,45,46]. Several vegetated corridors, such as the “Lomas” on the coast of the Atacama Desert, have been suggested [36,47]. These vegetated corridors were established and maintained by the humidity of the coastal fog known as “camanchaca”, and allowed the colonization and development of flora and fauna [36,46,47,48]. These corridors were already established at the time of the divergence of the northern and southern clades. *Mepraia* of the northern clade may have occupied these corridors to colonize north and south from a central origin (*M*. sp. Figure 3d–g), feeding on the fauna that occupied these foggy, isolated habitats. This kind of habitat has been present in coastal Atacama from the Middle Neogene [28,49,50]. Today, those three species mainly inhabit these fog oases, such as Paposo-Pan de Azúcar (*M. parapatrica*), Morro Moreno-Isla Santa María (*M*. sp.) and Alto Patache-Quebrada Vitor (*M. gajardoi*) (Figure 1a). This distribution pattern would reinforce the inter–intraspecific genetic structuring by hyperarid barriers even more.

PCO also affected the marine terraces due to rising sea levels, which were frequent in the late Pleistocene [51,52,53]. These changes in sea level and marine terraces had an impact on the structure of these habitats, probably separating populations on coastal islands [9] and causing the extinction of some populations that inhabited the littoral plains during high-sea-level periods. The extinctions caused by these changes left unoccupied habitats that could have been recolonized by ancient populations that were left in refuges. In particular, *M.* sp. could have originated from an ancestral refuge, but the diversification was very recent (~0.16 Mya), which is congruent with the estimated dates of changes that impacted the coast in this zone [52,54]. The extinction dynamics by sea-level changes left a very recent fingerprint on the genetic diversity of this probable cryptic lineage (*M.* sp.), even though it is the oldest lineage of the northern clade, and can also explain its small distribution range enclosed between Santa María Island and Morro Moreno, where only females have been collected (Figure 1a). 

We acknowledge the limitations of our results that only reflect the maternal history and that it is necessary to integrate nuclear genes to produce a more robust evolutionary history. However, these results provide valuable first spatio-temporal colonization evidence that can be tested with additional nuclear markers in the near future.

## 5. Conclusions

This study presents the first spatio-temporal analysis to show the colonization history of a blood-sucking insect in the Atacama Desert. After the last Andes uplift, *Mepraia* was definitively separated from its Argentinean sister group, which was reinforced by colder and more arid periods during the Pliocene. Subsequently, our results support the colonization of *Mepraia* from the Coquimban Biogeographical Province to the north. The current coastal distribution range of *Mepraia* nicely illustrates the border of the hyperarid core of the Atacama, which is a real-time picture of the effects of hyperaridity on the distribution of those insects. The colonization of those hyperarid environments should have occurred during the late Pliocene; later, by allopatry, a hyperarid cycle would have separated definitively the southern and northern clades (Figure 3). Today, the southern clade inhabits a more suitable environment, allowing it to occupy inland habitats, while the northern clade is pressured by the hyperarid core of the Atacama to inhabit a narrow coastal strip. In this coastal strip, there are a few fog oases that have a suitable habitat for surviving in this harsh environment. The isolation between these oases triggered population and species differentiation. The genetic structure is also likely affected by biotic factors, because *Mepraia* is a blood-sucking insect, but their effects would be less than those of the abiotic factors. Hyperarid barriers repeated several times during the PPCO, shaping the current diversity and genetic structure of *Mepraia.* The effect of these oscillations was greater in the Atacama Desert, where the diversity is higher, suggesting that it caused a major speciation process. This is because colonization should have occurred from the centre to the north and south following vegetated corridors during the humid periods of the Pleistocene, which were interrupted by long hyperarid periods. Finally, these results could provide relevant knowledge to understand the infection history of the protozoan *Trypanosoma cruzi* under a probable biogeographic scenario and help to understand the eco-epidemiology of Chagas disease in Chile.

## Figures and Tables

**Table 1:** Paired comparisons between different phylogenetic hypotheses using log (BF).

	S1	S2	S3	S4	S5
**S1**	*−8647.2*	38.5 *	23 *	18.1 *	8.8 *
**S2**	−38.5	*−8685.7*	−15.5	−20.4	−29.7
**S3**	−23	15.5	*−8670.2*	−4.9	−14.2
**S4**	−18.1	20.4	4.9	*−8665.3*	−9.3
**S5**	−8.8	29.7	14.2	9.3	*−8656*

S1–S5: different phylogenetic scenarios congruent with Figure 2. Italic numbers in the diagonal shows the log(ML) values for each scenario. Numbers with * indicate the best log(BF) values, with overwhelming support for scenario 1.

## Data Availability

The data presented in this study are available in the manuscript and in its Supplementary Material (Tables S1 and S2).

## Supplementary Materials

The following supporting information can be downloaded at: https://www.mdpi.com/article/10.3390/insects13050419/s1, Table S1: Sampling localities and accession numbers. Table S2: Historical and mutational parameters used in the coalescent simulations analysis.
