# Supplementary material for: Testing Phylogeographic Hypotheses in Mepraia (Hemiptera: Reduviidae) Suggests a Complex Spatio-Temporal Colonization in the Coastal Atacama Desert
Source: Insects. 2022 Apr 29;13(5):419. doi: 10.3390/insects13050419 (PMC9147758; doi:10.3390/insects13050419)
Supplement: Supplementary file 1 [file insects-13-00419-s001.zip › insects-1639704-supplementary.pdf]

Table S1. Sampling localities and accession numbers.

N: number of individuals sequenced. COIA: segment A of the cytochrome oxidase subunit I gene. COIB: segment B of the cytochrome oxidase subunit I gene. cyt b: cytochrome oxidase b gene segment. ND4: NADH dehydrogenase subunit 4 gene segment. Superscript letter in the accession number: [a] sequenced in [11], [b] sequenced in [7], [c] sequenced in [9]. Accession number without superscript indicates sequences obtained in this study. --- : segment not amplified.

					GenBank accession number			
**Locality**	**Latitude/longitude**	**N**	**Taxa**	**Species**	**COIB**	**COIA**	**cyt b**	**ND4**
La Rioja			Out	*T. eratyrusiformis*	MN117859[c]	GQ336898[a]	KC236979[b]	MZ348418
Corazones	18°28'47" S/70°19'27" W	13	C2	*M. gajardoi*	KC236913[b]	MZ348366	KC236946[b]	MZ348391
			C15	*M. gajardoi*	KC236914[b]	MZ348366	KC236947[b]	MZ348392
			C16	*M. gajardoi*	KC236915[b]	MZ348366	KC236948[b]	MZ348393
			C21	*M. gajardoi*	KC236916[b]	MZ348366	KC236949[b]	---
			C37	*M. gajardoi*	KC236917[b]	MZ348366	KC236950[b]	MZ348391
Caleta Vitor	18°45'45" S/ 70°20'34" W	13	V3	*M. gajardoi*	KC236918[b]	MZ348367	KC236951[b]	MZ348394
			V8	*M. gajardoi*	KC236919[b]	MZ348367	KC236952[b]	MZ348395
			V10	*M. gajardoi*	KC236920[b]	MZ348368	KC236953[b]	MZ348396
Caleta Camarones	19°12'16" S/ 70°16'08" W	13	Ca3	*M. gajardoi*	KC236921[b]	MZ348369	KC236954[b]	MZ348397
			Ca5	*M. gajardoi*	KC236922[b]	MZ348369	KC236955[b]	MZ348396
Río Seco	21°00'06" S/70°09'52" W	15	RS1	*M. gajardoi*	KC236923[b]	MZ348370	KC236956[b]	---
			RS12	*M. gajardoi*	KC236925[b]	MZ348371	KC236958[b]	---
			RS34	*M. gajardoi*	KC236926[b]	MZ348371	KC236959[b]	---
Caleta San Marcos	21°06'56" S/70°07'30" W	11	SM8	*M. gajardoi*	KC236924[b]	MZ348371	KC236957[b]	---
Santa María Island	23°25'51" S/70°36'31" W	16	ISMa1	*M.* sp	MN117861[c]	MZ348372	MN117875[c]	MZ348398
			ISMa7	*M.* sp	MN117862[c]	MZ348372	MN117876[c]	MZ348399
Santa María Continent	23°28'3" S/70°37'2" W	17	COSMA1	*M.* sp	MN117863[c]	MZ348373	MN117877[c]	MZ348400
			COSMA7	*M.* sp	MN117864[c]	MZ348373	MN117878[c]	MZ348401
Médano	24°36'51" S/70°33'31" W	11	Me34	*M. parapatrica*	KC236928[b]	---	KC236961[b]	MZ348405
Pan de Azúcar Island	26°10'27" S/70°39'58" W	26	IPA1	*M. parapatrica*	MN117865[c]	MZ348374	MN117879[c]	MZ348402
			IPA2	*M. parapatrica*	MN117866[c]	---	MN117880[c]	MZ348403
			IPA16	*M. parapatrica*	MN117867[c]	MZ348375	MN117881[c]	MZ348402
			IPA20	*M. parapatrica*	MN117868[c]	MZ348376	MN117882[c]	MZ348403
			IPA36	*M. parapatrica*	MN117869[c]	MZ348376	MN117883[c]	MZ348402
Pan de Azúcar Continent	26° 8'34" S/ 70°40'10" W	23	COPA3S2	*M. parapatrica*	MN117870[c]	MZ348377	MN117884[c]	MZ348404
Caleta Zenteno	26°51'08" S/70°48'36" W	16	Z8	*M. parapatrica*	KC236929[b]	MZ348378	KC236962[b]	MZ348406

			Z12	*M. parapatrica*	KC236930[b]	MZ348378	KC236963[b]	MZ348407
Inca de Oro	26°48'15" S/69°57'14" W	9	Inca1	*M. spinolai*	MN117871[c]	---	MN117885[c]	MZ348408
			Inca2	*M. spinolai*	MN117872[c]	---	MN117886[c]	MZ348408
			Inca11	*M. spinolai*	MN117873[c]	---	MN117887[c]	MZ348409
Llanos de Challe	28°08'52" S/71°04'32" W	8	Lla1	*M. spinolai*	KC236931[b]	MZ348379	KC236964[b]	---
			Lla3	*M. spinolai*	KC236932[b]	MZ348379	KC236965[b]	---
			Lla4	*M. spinolai*	KC236933[b]	MZ348380	KC236966[b]	MZ348410
			Lla13	*M. spinolai*	KC236934[b]	MZ348381	KC236967[b]	MZ348411
Alto del Carmen	28°43'21" S/70°31'02" W	14	AC2	*M. spinolai*	KC236935[b]	MZ348382	KC236968[b]	MZ348412
			AC11	*M. spinolai*	KC236936[b]	MZ348383	KC236969[b]	MZ348412
Caleta Toro	30°44'30" S/71°42'05" W	14	T1	*M. spinolai*	KC236937[b]	MZ348384	KC236970[b]	MZ348414
Monte Patria	30°51'16" S/70°41'51" W	14	Mp1	*M. spinolai*	KC236939[b]	---	KC236972[b]	MZ348413
			Mp4	*M. spinolai*	KC236940[b]	---	KC236973[b]	MZ348413
RN Las Chinchillas	31°30'28" S/ 71°06'19" W	11	RNCh26	*M. spinolai*	KC236941[b]	MZ348385	KC236974[b]	MZ348415
			RNCh176	*M. spinolai*	KC236942[b]	MZ348386	KC236975[b]	MZ348415
			RNCh345	*M. spinolai*	KC236943[b]	MZ348387	KC236976[b]	MZ348416
San Felipe	32°51'24" S/70°29'13" W	4	SF1	*M. spinolai*	MN117874[c]	MZ348388	MN117888[c]	---
Til Til	33°06'19" S/70°55'53" W	11	Til1	*M. spinolai*	KC236944[b]	MZ348389	KC236977[b]	MZ348417
			Til32	*M. spinolai*	KC236945[b]	MZ348390	KC236978[b]	MZ348417

Table S2. Historical and mutational parameters used in the coalescent simulations analysis.

Parameter	Prior distribution	
	Type	Initial-Final interval
Ne	Uniform	200,000 - 450,000
t1	Uniform	1300000 - 1600000
t2	Uniform	1700000 - 2100000
t3	Uniform	2000000 - 2600000
Mean mutation rate	Uniform	1.00E-8 - 5.00E-8
